# Localisation of Sensor Nodes with Hybrid Measurements in Wireless Sensor Networks ^†^

**DOI:** 10.3390/s16071143

**Published:** 2016-07-22

**Authors:** Muhammad W. Khan, Naveed Salman, Andrew H. Kemp, Lyudmila Mihaylova

**Affiliations:** 1 School of Electronic and Electrical Engineering, University of Leeds, Leeds LS2 9JT, UK; a.h.kemp@leeds.ac.uk; 2 Department of Automatic Control and Systems Engineering, Sheffield University, Sheffield S1 3JD, UK; n.salman@sheffield.ac.uk (N.S.); l.s.mihaylova@sheffield.ac.uk (L.M.)

**Keywords:** hybrid localisation, received signal strength, angle of arrival, generalised pattern search

## Abstract

Localisation in wireless networks faces challenges such as high levels of signal attenuation and unknown path-loss exponents, especially in urban environments. In response to these challenges, this paper proposes solutions to localisation problems in noisy environments. A new observation model for localisation of static nodes is developed based on hybrid measurements, namely angle of arrival and received signal strength data. An approach for localisation of sensor nodes is proposed as a weighted linear least squares algorithm. The unknown path-loss exponent associated with the received signal strength is estimated jointly with the coordinates of the sensor nodes via the generalised pattern search method. The algorithm’s performance validation is conducted both theoretically and by simulation. A theoretical mean square error expression is derived, followed by the derivation of the linear Cramer-Rao bound which serves as a benchmark for the proposed location estimators. Accurate results are demonstrated with 25%–30% improvement in estimation accuracy with a weighted linear least squares algorithm as compared to linear least squares solution.

## 1. Introduction

Localisation of wireless devices has become exceedingly important in many applications. These include logistics, robotics and surveillance [1]. Range based approaches are favoured for accurate localisation. Two main groups of techniques for estimating the range between sensor nodes are based on the time of arrival (ToA) and the received signal strength (RSS) approach. Location coordinates of nodes can also be estimated by utilising the angle of the impinging signal, this is known as the angle of arrival (AoA) technique. Individual analysis and optimisation of these techniques have been widely studied [2,3]. For range based models, the localisation problem can be solved via high complexity maximum likelihood techniques [4]. A low complexity linear least squares (LLS) approach has also been proposed for ToA based systems [5], its performance is analysed and enhanced in [6]. Similar approaches are adapted for RSS based localisation [3]. On the other hand, the AoA of the signal can be estimated using an array of antennas as in [7] or a rotating beam of radiation [8], and using techniques such as Multiple Signal Classification [9] or estimation of signal parameters via rotational invariance techniques [10].

### Related work

With the increasing demand of high accuracy positioning, hybrid signal based localisation is becoming more and more popular. A highly celebrated hybrid AoA-ToA signal model is proposed in [11] which produces biased estimates of the location vector. An improved and unbiased version of which is presented in [12] where a weighted solution is presented. In [13], an angle based localisation model is presented, where the angles are derived from RSSs from different beacons. The algorithm works on a fingerprinting based approach and will fail to perform when the network scenario changes. In [14], the bearing measurements are utilised together with range-difference measurements obtained from time difference of arrival of the signal from multiple sensors. Together with geometric constraint on measurements errors an improved localisation algorithm is proposed for static target nodes. A two step range and angle based positioning is presented in [15], in which the range estimation in the first step is utilised with differential angle measurement obtained in the second step. A cooperative version of hybrid localisation based on ToA and AoA is proposed in [16], which achieves a very high accuracy at the cost of high computational load. Moreover, non-line of sight components of the signal are detected by an iterative algorithm which is based on the incoming hybrid signals in [17]. In [18], positioning and tracking of people is performed using the extended Kalman filter based on time difference of arrival and AoA. Positioning of people is an important application of WSN and has a vital significance in health care systems [19]. In contrast to the mentioned literature, our work is based on the noise covariance of the hybrid measurements and produces completely unbiased estimates of the unknown vector while considering a realistic assumption of unknown path-loss exponent (PLE) vector.

This paper proposes a LLS estimator based on a hybrid AoA-RSS measurement model which produces unbiased estimates of the unknown vector of location coordinates. Furthermore, in order to improve accuracy, the link quality between the anchor node (AN) and the target node (TN) is considered, which is provided by the covariance matrix. Thus, the covariance matrix is first derived and a weighted linear least squares (WLLS) estimator is proposed. Moreover it is noted that different combinations of ANs perform optimally in different sections of the network. As a result, the network can be decomposed into different zones where each zone corresponds to a unique combination of ANs. Hence, a technique that selects this optimal set of ANs for different zones is proposed. In case of ranging via RSS the correct knowledge of PLE associated with each link is required. In most studies the PLEs are assumed to be known, which is an oversimplification of real conditions. Some recent studies jointly estimate the location coordinates and the PLE for localisation [20,21] for RSS measurements only. However, these studies assume the same PLE for every AN-TN link, which is not a valid assumption for real data. In contrast with [20,21], in this paper, we assume an unknown and a different PLE value for each communication link and we propose a novel PLE estimator, based on the generalised pattern search algorithm.

The main contributions of this paper are as follows:

- A new unbiased observation model for localisation of static nodes is developed based on hybrid measurements, namely angle-of-arrival and received-signal-strength data.
- A WLLS framework based on the noise covariance of the signal is presented.
- The mathematical derivation of unbiasness and unbias constant is given.
- A two step AN selection technique is presented which further improves the performance.
- Theoretical results for the mean square error (MSE) are derived.
- Joint PLE and sensor node coordinates estimation is proposed via generalised pattern search (A dynamic version was presented in [22] for mobile nodes).
- The linear Cramer-Rao bound (LCRB) is derived for the WLLS algorithm.
- A more practical scenario for simulation is considered where the TNs are situated inside as well as outside the convex hull defined by ANs.

The rest of the paper is organized as follows: Section 2 presents the problem statement and the unbiased system model. The WLLS algorithm is proposed in Section 3. In Section 4 the two step AN selection strategy is presented and the theoretical MSE for LLS is derived. A PLE estimator via the generalised pattern search is proposed in Section 5. The LCRB is derived in Section 6. Finally, in Section 7, we discuss the simulation results which are followed by conclusions in Section 8.

## 2. System Model

The following notations are introduced: $R^{n}$ and $Z^{n}$ are the sets of *n* dimensional real numbers and integers respectively. Also, $N(\mu ,\sigma^{2})$ and $U\left[U_{min},\;U_{max}\right]$ denotes the normal distribution with mean *μ* and variance $\sigma^{2}$ and uniform distribution between $U_{min}$ and $U_{max}$, respectively. A two dimensional network is considered consisting *N* ANs with known locations i.e., $u_{i}={\left[x_{i},y_{i}\right]}^{T}$ $\left(u_{i}\in R^{2}\right)$ for i=1,…,N and a TN which has unknown coordinates i.e., $u={\left[x,y\right]}^{T}$ $\left(u\in R^{2}\right)$. Unlike conventional trilateration, in hybrid systems the AN does not define a circle, but rather defines a line. At one end of the line the AN is situated with known position while the TN is situated at the opposite end for which the coordinates are to be estimated. If the slope (AoA) and the magnitude (RSS) information of this line is available, then the TN coordinates can be easily determined using trigonometric equations. The AN receives a signal with line of sight and non-line of sight components. The line of sight and non-line of sight detection is beyond the scope of this paper. Readers are referred to [23,24] for line of sight/non-line of sight detection and mitigation techniques. This section presents the angle of arrival-received signal strength measurement for localisation which serves as a base for the rest of the paper. Let $\left(x_{i},y_{i}\right)$ be the coordinates of *i*th AN then the *x* and *y* coordinates of the TN in the presence of both range and angle estimates are given by [25]

(1) $$\hat{x}=x_{i}+{\hat{d}}_{i}\cos{\hat{\theta }}_{i}\delta_{i}$$

(2) $$\hat{y}=y_{i}+{\hat{d}}_{i}\sin{\hat{\theta }}_{i}\delta_{i}$$

where ${\hat{d}}_{i},$ ${\hat{\theta }}_{i}$ and $\delta_{i}$ represent range measurements, angle measurement and the unbiasing constant, respectively. The angle measurement ${\hat{\theta }}_{i}$ is given by

(3) $${\hat{\theta }}_{i}=\arctan\left[\frac{\left(y-y_{i}\right)}{\left(x-x_{i}\right)}\right]+m_{i}+\phi_{i}$$

where $m_{i}$ represents the zero mean Gaussian noise in angle estimate i.e., $m_{i}∼\left(N\left(0,\sigma_{m_{i}}^{2}\right)\right)$ and $\phi_{i}$ represents the angular spread caused by the non-line of sight signal which can be statistically described as a Gaussian random variable and can be calculated from experimental data [26]. In this paper, we restrict our attention to line of sight signals only. The non-line of sight detection/mitigation for angle estimation is studied in [27,28]. Equation (3) can be written in vector form as

(4) $$\hat{\theta }=f\left(u\right)+m$$

where $\hat{\theta }={\left[{\hat{\theta }}_{1},\ldots ,{\hat{\theta }}_{N}\right]}^{T}$, $f\left(u\right)={\left[\arctan\left[\left(y\;-\;y_{1}\right)\;/\;\left(x\;-\;x_{1}\right)\right],\ldots ,\arctan\left[\left(y\;-\;y_{N}\right)\;/\;\left(x\;-\;x_{N}\right)\right]\right]}^{T}$, $m={\left[m_{1},\ldots ,m_{N}\right]}^{T}$ is the noise vector and ${\left(.\right)}^{T}$ represents the transpose operator.

The range measurement, ${\hat{d}}_{i}$, is extracted from the path-loss $L_{i}$.

(5) $$L_{i}=L_{0}+10\alpha_{i}\log_{10}d_{i}+w_{i}$$

where $L_{0}$ is the path-loss at reference distance $d_{0},$ normally taken as 1m for indoor scenarios and is dependent on antenna characteristics, $w_{i}$ is the zero mean Gaussian random variable characterizing the shadowing effects i.e., $w_{i}∼\left(N\left(0,\sigma_{w_{i}}^{2}\right)\right),$ $\alpha_{i}$ represents the PLE associated with *i*th AN with value range from 2–5, depending on the environment, The observed path-loss $z_{i}$ from $d_{0}$ to $d_{i}$ is given as $L_{i}-L_{0}$, and can be represented as

(6) $${\hat{z}}_{i}=\gamma \alpha_{i}\ln d_{i}+w_{i}$$

where $\gamma =\frac{10}{\ln10}$. The distance estimates from Equation (6) can be obtained as [29]

(7) $${\hat{d}}_{i}=d_{i}\exp\left(\frac{w_{i}}{\gamma \alpha_{i}}\right)\kappa_{i}$$

where $\kappa_{i}$ is the unbiasing constant for RSS measurement only and is given by $\kappa_{i}=\exp\left(-\sigma_{w_{i}}^{2}/2{\left(\gamma \alpha_{i}\right)}^{2}\right)$. Equation (7) can be represented in vector form as $\hat{d}=d⊙\left[\exp\left(\frac{1}{\gamma \alpha_{i}}\left(w\right)\right)\right]⊙\kappa$, where $\hat{d}={\left[{\hat{d}}_{1},\ldots ,{\hat{d}}_{N}\right]}^{T}$, $d={\left[d_{1},\ldots ,d_{N}\right]}^{T},$ $\kappa =\left[\kappa_{1},\ldots ,\kappa_{N}\right]$ and $w={\left[w_{1},\ldots ,w_{N}\right]}^{T}$ represents the shadowing component vector. The mathematical symbol ⊙ represents the Schur product. The unbiasing constant for AoA-RSS signal is given by

(8) $$\delta_{i}=\exp\left(\frac{\sigma_{m_{i}}^{2}}{2}-\frac{\sigma_{w_{i}}^{2}}{2{\left(\gamma \alpha_{i}\right)}^{2}}\right)$$

which can be represented in vector form as $\delta ={\left[\delta_{1},\ldots ,\delta_{N}\right]}^{T}$.

Thus, Equations (1) and (2) can be written in matrix form as

(9) $$\hat{b}=\mathrm{Au}+q$$

where

(10) $$\begin{array}{c} A=\mathrm{diag}\left[e_{N},e_{N}\right]\in \;R^{2N\times 2},\;u={\left[x,y\right]}^{T}\;\in \;R^{2\times 1}\\ \hat{b}={\left[\hat{b}(x),\hat{b}(y)\right]}^{T}\;\in \;R^{2N\times 1} \end{array}$$

and $e_{N}$ is a column vector of *N* ones and q is the noise vector with zero mean vector and covariance $C\left(u\right).$ In Equation (10), $\hat{b}\left(x\right)$ and $\hat{b}\left(y\right)$ are given as

$$\begin{array}{cc} \hat{b}\left(x\right) & ={\left[x_{1}+{\hat{d}}_{1}\cos{\hat{\theta }}_{1}\delta_{1},\ldots ,x_{N}+{\hat{d}}_{N}\cos{\hat{\theta }}_{N}\delta_{N}\right]}^{T}\in \;R^{N\times 1}\\ \hat{b}\left(y\right) & ={\left[y_{1}+{\hat{d}}_{1}\sin{\hat{\theta }}_{1}\delta_{1},\ldots ,y_{N}+{\hat{d}}_{N}\sin{\hat{\theta }}_{N}\delta_{N}\right]}^{T}\in \;R^{N\times 1} \end{array}$$

Then the LLS solution is given by

(11) $$\hat{u}=A^{†}\hat{b}$$

where $A^{†}$ is the Moore–Penrose pseudoinverse of matrix A and is given by $A^{†}={\left(A^{T}A\right)}^{-1}A^{T}.$

Justification For $\delta_{i}$:

Without considering the unbiasing constant, Equation (11) produces biased estimates of the unknown vector u. We now show mathematically that the unbiasing constant is imperative for unbias estimation. The bias of the LLS technique is given by

(12) $$\mathrm{Bias}=A^{†}{\left[\epsilon \left(x\right)\epsilon \left(y\right)\right]}^{T}$$

where $\epsilon \left(x\right)\;=\;E\left[\hat{b}\left(x\right)\right]\;-\;b\left(x\right)$ and $\epsilon \left(y\right)\;=\;E\left[\hat{b}\left(y\right)\right]\;-\;b\left(y\right)$. $b\left(x\right)$ and $b\left(y\right)$ represents noise free observation. Then the *i*th term of $\epsilon \left(x\right)$ and $\epsilon \left(y\right)$ is given by

(13) $$\begin{array}{cc} \epsilon {\left(x\right)}_{i} & \;=\;E_{m_{i},w_{i}}\;\left[d_{i}\exp\left(\;\frac{w_{i}}{\gamma \alpha_{i}}\;\right)\cos\left(\theta_{i}\;+\;m_{i}\right)\;-\;d_{i}\cos\theta_{i}\right] \end{array}$$

(14) $$\begin{array}{cc} \epsilon {\left(y\right)}_{i} & \;=\;E_{m_{i},w_{i}}\;\left[d_{i}\exp\left(\;\frac{w_{i}}{\gamma \alpha_{i}}\;\right)\sin\left(\theta_{i}+m_{i}\right)\;-\;d_{i}\sin\theta_{i}\right] \end{array}$$

where $E_{\left(.\right)}$ represents the mathematical expectation operation. Equations (13) and (14) are reduced to

(15) $$\begin{array}{cc} \epsilon {\left(x\right)}_{i} & =d_{i}\cos\theta_{i}\left(\exp\left(-\frac{\sigma_{m_{i}}^{2}}{2}+\frac{\sigma_{w_{i}}^{2}}{2{\left(\gamma \alpha_{i}\right)}^{2}}\right)-1\right) \end{array}$$

(16) $$\begin{array}{cc} \epsilon {\left(y\right)}_{i} & =d_{i}\sin\theta_{i}\left(\exp\left(-\frac{\sigma_{m_{i}}^{2}}{2}+\frac{\sigma_{w_{i}}^{2}}{2{\left(\gamma \alpha_{i}\right)}^{2}}\right)-1\right) \end{array}$$

Thus, we use $\delta_{i}$ in Equations (1) and (2) to reduce Equations (15) and (16) to zero and consequently Equation (12) to zero. The proof is given in Appendix A.

## 3. Weighted Linear Least Squares Algorithm

The performance of LLS can be improved by utilising the communication link quality between ANs and the TN. Thus links with larger noise are given small weights as compared with links with small noise. This link quality is provided by the covariance matrix. In this section, we exploit the covariance matrix and propose a WLLS solution which is obtained by minimizing the cost function.

(17) $$\varepsilon_{WLLS}\left(\hat{u}\right)={\left(\hat{b}-Au\right)}^{T}C^{-1}\left(u\right)\left(\hat{b}-Au\right)$$

where $C\left(u\right)$ is the covariance matrix given by $C\left(u\right)=E_{m,w}\left[\left(\hat{b}-b\right){\left(\hat{b}-b\right)}^{T}\right]$. The matrix $C\left(u\right)$ can be partitioned into sub-matrices as

(18) $$C\left(u\right)=\left[\begin{array}{cc} C\left(x\right) & C\left(xy\right)\\ C\left(xy\right) & C\left(y\right) \end{array}\right]\;\in \;R^{2N\times 2N}$$

The sub-matrices in Equation (18) are given as follows

(19) $$C\;\left(x\right)\;=\;E_{m,w}\;\;\left[\;\left(\;\hat{b}\;\left(x\right)\;-\;b\;\left(x\right)\;\right){\left(\;\hat{b}\;\left(x\right)\;-\;b\;\left(x\right)\;\right)}^{\;\;T}\right]\in R^{N\times N}$$

(20) $$C\;\left(y\right)\;=\;E_{m,w}\;\;\left[\;\left(\;\hat{b}\;\left(y\right)\;-\;b\;\left(y\right)\;\right){\left(\;\hat{b}\;\left(y\right)\;-\;b\;\left(y\right)\;\right)}^{\;\;T}\right]\in R^{N\times N}$$

(21) $$\;C\;\left(xy\right)\;=\;E_{m,w}\;\;\left[\;\left(\;\hat{b}\;\left(x\right)\;-\;b\;\left(x\right)\;\right){\left(\;\hat{b}\;\left(y\right)\;-\;b\;\left(y\right)\;\right)}^{\;\;T}\right]\;\in \;R^{N\times N}$$

Then for AoA-RSS measurement Equations (19)–(21) reduces to Equations (22)–(24) for i=j and to 0 for i≠j.

(22) $$C{\left(x\right)}_{ii}=\frac{d_{i}^{2}}{2}\kappa_{i}+\frac{d_{i}^{2}}{2}\cos\left(2\theta_{i}\right){\bar{\kappa }}_{i}-{\left(d_{i}\cos\theta_{i}\right)}^{2}$$

(23) $$C\left(y\right){}_{ii}=\frac{d_{i}^{2}}{2}\kappa_{i}-\frac{d_{i}^{2}}{2}\cos\left(2\theta_{i}\right){\bar{\kappa }}_{i}-{\left(d_{i}\sin\theta_{i}\right)}^{2}$$

(24) $$C{\left(xy\right)}_{ii}\;=\;d_{i}^{2}\cos\theta_{i}\sin\theta_{i}\left({\bar{\kappa }}_{i}-1\right)$$

$$C\left(x\right){}_{ij}=0,\;C{\left(y\right)}_{ij}=0,\;C{\left(xy\right)}_{ij}=0$$

where $\kappa_{i}=\exp\left(\frac{\sigma_{w_{i}}^{2}}{{\left(\gamma \alpha_{i}\right)}^{2}}+\sigma_{m_{i}}^{2}\right)$, ${\bar{\kappa }}_{i}=\exp\left(\frac{\sigma_{w_{i}}^{2}}{{\left(\gamma \alpha_{i}\right)}^{2}}-\sigma_{m_{i}}^{2}\right)$ and the notation $T_{ij}$ refers to the element at the *i*th row and *j*th column of any matrix T.

The elements of $C\left(u\right)$ in Equation (18) depends on the real values of distances and angles, which are not available. Thus, their estimated values are used to calculate the covariance matrix. Now the WLLS solution can be obtained as follows,

(25) $${\hat{u}}_{WLLS}=A^{‡}b^{‡}$$

where $A^{‡}={\left[A^{T}C^{-1}\left(\hat{u}\right)A\right]}^{-1}A^{T}$ and ${\hat{b}}^{‡}=C^{-1}\left(\hat{u}\right)\hat{b}$.

Further performance improvement can be attained by optimal selection of ANs described in the next section.

## 4. Two Step Optimal AN Selection

Due to the unequal error associated with different ANs, some ANs may actually deteriorate the positioning accuracy. These ANs may be positioned at a large distance from the TN or they may receive signal through multiple paths or it may have a poor geometric dilution of precision. This scenario is more obvious in a network where some TNs are outside the convex hull defined by the ANs. Thus, for different TNs in a network there exists an optimal subset of ANs that will produce better estimates than estimates produced while using all ANs. In this section, we present a two step optimal subset selection scheme. A pre-processing step, called zone detection, selects different subsets of ANs for different TNs followed by localisation using the optimal subset of ANs.

**Step I: Zone Detection:** During this pre-processing step, the whole network is divided into a grid. The complexity of this step depends on the resolution of the grid and the total number of ANs. However, this step needs to be performed only once. Each point on the grid acts a pseudo-TN. For each of these pseudo-TN, the localisation error is calculated for all combinations of ANs using the theoretical MSE presented in the next subsection. The combination that shows the lowest MSE is selected as an optimal combination of anchor nodes for that point. Thus using this technique a particular combination is selected for different points on the grid. In this way the whole network is divided into different regions called zones, where each zone has its own optimal subset of ANs that shows that the minimum MSE during localisation in the next step.

The theoretical MSE of AoA-RSS signal model: The derivation of the theoretical MSE for LLS is performed as follows.

(26) $$\mathrm{MSE}\left(\hat{u}\right)=\mathrm{Tr}\left\{E_{w,m}\left[\left(\hat{u}-u\right){\left(\hat{u}-u\right)}^{T}\right]\right\}$$

where $\hat{u}$ is the estimate of the location vector, u is $A^{†}b$ is the ground truth and Tr(.) represents the trace operator. Equation (26) can be simplified as follows

(27) $$\begin{array}{ll} MSE\left(u\right) & =Tr\left\{E_{w,m}\left[\left(A^{†}\hat{b}-A^{†}b\right){\left(A^{†}\hat{b}-A^{†}b\right)}^{T}\right]\right\}\\ & =Tr\left\{E_{w,m}\;\left[\;\left(\;A^{†}\hat{b}\;-\;A^{†}b\right)\;\left(\;\hat{b}{\left(A^{†}\right)}^{T}\;\;\;-\;b{\left(A^{†}\right)}^{T}\;\right)\;\right]\;\right\}\\ & =Tr\left\{A^{†}E_{w,m}\left[\left(\hat{b}-b\right)\left(\hat{b}-b\right)\right]{\left(A^{†}\right)}^{T}\right\}\\ & =Tr\left\{A^{†}C\left(\hat{u}\right){\left(A^{†}\right)}^{T}\right\} \end{array}$$

Thus in the offline stage, the combination of ANs minimizing Equation (27) for a particular grid point is selected as the optimal AN subset.

**Step II: Localisation with optimal combination of ANs:** The second step is also two fold. Firstly, a rough estimate of the location of the TN is obtained using all ANs. This rough estimate is necessary to detect the zone where the TN belongs. Once the zone is detected, the location of this TN is refined by localising it again, this time using the optimal combination of ANs for its respective zone.

## 5. Estimation of Unknown PLE

In order to estimate the distance from the target node to the anchor node, the correct knowledge of PLE associated with each link is necessary. Most of the localisation techniques assume that the PLE is known and same for all links. However, even a small error in the PLE vector produces a significant error in the estimated location. In contrast with the common localisation techniques, in this paper we consider the case when the PLE is unknown and has a different value for every link. A new PLE-generalised pattern search algorithm is proposed. For the observation vector $\hat{b}$, given by Equation (10), the cost function $\Psi \left(u,\alpha \right)$ with unknown PLE vector and TN’s coordinates vector u is given by

(28) $$\Psi \left(u,\alpha \right)=∥Au-\hat{b}{∥}^{2}$$

where ***α*** is the PLE vector given by, $\alpha =\left[\alpha_{1},\ldots ,\alpha_{N}\right].$ In Equation (28), ***α*** and u are unknown. The LLS solution to u is given by Equation (11) and after replacing it in Equation (28) gives [30]

(29) $$\Psi \left(\alpha \right)=\left[\left[b\left(x\right)\;b\left(y\right)\right]\left(I_{2N}-AA^{†}\right){\left[b\left(x\right)\;b\left(y\right)\right]}^{T}\right]$$

Equation (29) has only one unknown i.e. the vector α. In Equation (29)

(30) $$b\left(x\right)\;=\;{\left[\exp\left(\;\frac{{\hat{z}}_{1}}{\gamma \alpha_{1}}\;\right)\cos{\hat{\theta }}_{1}\delta_{1},\ldots ,\exp\left(\;\frac{{\hat{z}}_{N}}{\gamma \alpha_{N}}\;\right)\cos{\hat{\theta }}_{N}\delta_{N}\right]}^{T}$$

and

(31) $$b\left(y\right)\;=\;{\left[\exp\left(\;\frac{{\hat{z}}_{1}}{\gamma \alpha_{1}}\;\right)\sin{\hat{\theta }}_{1}\delta_{1},\ldots ,\exp\left(\;\frac{{\hat{z}}_{N}}{\gamma \alpha_{N}}\;\right)\sin{\hat{\theta }}_{N}\delta_{N}\right]}^{T}$$

and $I_{2N}$ is an identity matrix of dimension 2N. The solution to Equation (29) is given by

(32) $$\hat{\alpha }=\arg\min_{\alpha }\left\{\Psi \left(\alpha \right)\right\}$$

Equation (32) can be solved by a brute force search method which is computationally expensive as the cost function has to be evaluated at all possible values of ***α***. For a large number of nodes the brute force search method becomes impractical. Computationally more efficient the generalise pattern search method is therefore used to minimise Equation (32). The generalised pattern search for minimisation of Equation (32) is presented in the next subsection.

### Generalised Pattern Search

The generalised pattern search belongs to a family of derivative-free optimisation techniques. Starting from an initial guess for $\alpha_{0}\in \left[2,5\right]$ for most environments [31] and an initial step size $\Delta_{0}$, The generalised pattern search iteratively updates $\alpha_{k}$ such that $\Psi \left(\alpha_{k+1}\right)<\Psi \left(\alpha_{k}\right)$, where $\alpha_{k}$ represents the value at *k*th iteration. Each update evaluates the cost function Equation (32) at a point on the mesh, with the updated point closer to the minimum of $\Psi \left(\alpha \right).$

Each iteration consists of a search (optional) and a poll step. At each of these steps the cost function is evaluated on a mesh $M_{k}$, centred at $\alpha_{k}$ and defined by D, a finite set of direction which positively spans $R^{N}$. The directions can be chosen by any strategy. However each direction ${\bar{d}}_{j}\;\forall \;j=1,\ldots ,q$ must be a product of $Gz_{j},$ where *q* is the cardinality of D, $G\in R^{N\times N}$ is a non-singular generating matrix which for the present problem is $G=\frac{1}{\nu }I_{N}$ for ν>1 and $z_{j}\in Z^{N}$ is an integer vector. These conditions are necessary to the convergence theory [32]. Let $Z\in Z^{N\times q}$ denotes a matrix whose columns are $z_{j}\forall j=1,\ldots ,q,$ then D is represented as the product of GZ and the mesh centred at $\alpha_{k}$ is given by

(33) $$M_{k}=\left\{\alpha_{k}+\Delta_{k}\mathrm{Dz}:\;z\in Z^{q}\right\}$$

At the *k*th poll, the cost function is evaluated at neighbouring poll points given by $P_{k}=\left\{\alpha_{k}+\Delta_{k}\bar{d},\;\bar{d}\in D_{k}\right\}$. Thus at $\left(k+1\right)$th iteration if the cost function value i.e., $\Psi \left(\alpha_{k+1}\right)$ is lower than $\Psi \left(\alpha_{k}\right)$ then the step size is increased by $\Delta_{k+1}=\xi \Delta_{k}$ for any scalar ξ>1 and $\alpha_{k+1}$ is accepted i.e., $M_{k+1}$ is centred at $\alpha_{k+1}$. Otherwise if $\Psi \left(\alpha_{k+1}\right)>\Psi \left(\alpha_{k}\right)$ for all the poll points then the step size is decreased by $\Delta_{k+1}=\frac{1}{\xi }\Delta_{k}$ and $\alpha_{k+1}=\alpha_{k}.$ The algorithm is repeated until a stopping condition is reached e.g., $\Psi \left(\alpha_{k+1}\right)-\Psi \left(\alpha_{k}\right)<\tau$, where *τ* is some small value. The generalised pattern search for PLE estimation is presented in Algorithm 1.

**Algorithm 1:** Generalised Pattern Search  **for**
k=1,…   i. Initialize $\alpha_{0}\in \left[2\;5\right]$, $\Delta_{0},$
τ,ξ,ν.   ii. Evaluate $\Psi \left(\alpha_{k+1}\right)$ with all poll points from poll set $\left\{\alpha_{k}+\Delta_{k}\bar{d},\bar{d}\in D\right\}$.   iii-a. If improved poll point is found, accept $\alpha_{k+1},$ set $\Delta_{k+1}=\xi \Delta_{k}$.   iii-b. If improved poll point cannot be found, set $\alpha_{k+1}=\alpha_{k}$, set $\Delta_{k+1}=\frac{\Delta_{k}}{\xi }$.   Repeat until $\Psi \left(\alpha_{k+1}\right)-\Psi \left(\alpha_{k}\right)<\tau$.  **end**

## 6. Linear Cramer-Rao Bound

The Cramer-Rao bound characterizes the best possible accuracy of an unbiased estimator. The conventional localisation Cramer-Rao bound is based on individual readings from ANs. Conversely, the LLS and WLLS formulation is based on observation vector $\hat{b}$. In order to lower bound the performance of WLLS, we derive the LCRB in this section. The maximum accuracy of the two dimensional localisation is characterized by the MSE bound:

(34) $$\mathrm{MSE}\left(u\right)\geq \frac{{\left[I\left(u\right)\right]}_{11}+{\left[I\left(u\right)\right]}_{22}}{\det\left[I\left(u\right)\right]}$$

where $\left[I\left(u\right)\right]$ is the Fisher information matrix (FIM) whose elements are given by Equation (35) [30].

(35) $${\left[I\left(u\right)\right]}_{ij}=\left[\frac{\partial \mu \left(u\right)}{\partial u_{i}}\right]C^{-1}\left(u\right)\left[\frac{\partial \mu \left(u\right)}{\partial u_{j}}\right]+\frac{1}{2}Tr\left[\left(C^{-1}\left(u\right)\frac{\partial C\left(u\right)}{\partial u_{i}}C^{-1}\left(u\right)\frac{\partial C\left(u\right)}{\partial u_{j}}\right)\right]$$

where $\mu \left(u\right)=\left[x_{1}+d_{1}\cos\theta_{1}\right],\ldots ,x_{N}+d_{N}\cos\theta_{N},$ $\ldots ,y_{1}+d_{1}\sin\theta_{1},$ $\ldots ,{\left.y_{N}+d_{N}\sin\theta_{N}\right]}^{T}$ is the mean of the observation vector. The derivatives are obtained as follows:

(36) $$\frac{\partial \mu }{\partial x}={\left[1_{1},1_{2},\cdots 1_{N},0_{1},0_{2},\cdots 0_{N}\right]}^{T}$$

(37) $$\frac{\partial \mu }{\partial y}={\left[0_{1},0_{2},\cdots 0_{N},1_{1},1_{2},\cdots 1_{N}\right]}^{T}$$

(38) $$\frac{\partial C{\left(x\right)}_{{}_{ii}}}{\partial x}=\left(x-x_{i}\right)\kappa_{i}+\left[\left(y-y_{i}\right)\sin2\theta_{i}+\left(x-x_{i}\right)\cos2\theta_{i}\right]{\bar{\kappa }}_{i}-2\left(x-x_{i}\right)$$

(39) $$\frac{\partial C{\left(x\right)}_{{}_{ii}}}{\partial y}=\;\left(y-y_{i}\right)\kappa_{i}+\left[\left(y-y_{i}\right)\cos2\theta_{i}-\left(x-x_{i}\right)\sin2\theta_{i}\right]{\bar{\kappa }}_{i}$$

(40) $$\frac{\partial C{\left(y\right)}_{{}_{ii}}}{\partial x}=\left(x-x_{i}\right)\kappa_{i}-\left[\left(y-y_{i}\right)\sin2\theta_{i}+\left(x-x_{i}\right)\cos2\theta_{i}\right]{\bar{\kappa }}_{i}$$

(41) $$\frac{\partial C{\left(y\right)}_{{}_{ii}}}{\partial y}=\left(y-y_{i}\right)\kappa_{i}-\left[\left(y-y_{i}\right)\cos2\theta_{i}-\left(x-x_{i}\right)\sin2\theta_{i}\right]{\bar{\kappa }}_{i}-2\left(y-y_{i}\right)$$

(42) $$\frac{\partial C{\left(xy\right)}_{{}_{ii}}}{\partial x}=\left[\sin2\theta_{i}\left(x-x_{i}\right)-\cos2\theta_{i}\left(y-y_{i}\right)\right]\left[{\bar{\kappa }}_{i}-1\right]$$

(43) $$\frac{\partial C{\left(xy\right)}_{{}_{ii}}}{\partial y}=\left[\cos2\theta_{i}\left(x-x_{i}\right)+\sin2\theta_{i}\left(y-y_{i}\right)\right]\left[{\bar{\kappa }}_{i}-1\right]$$

The derivation of Equation (38) to Equation (43) are given in Appendix B.

## 7. Simulation Results

**Figure 1:**
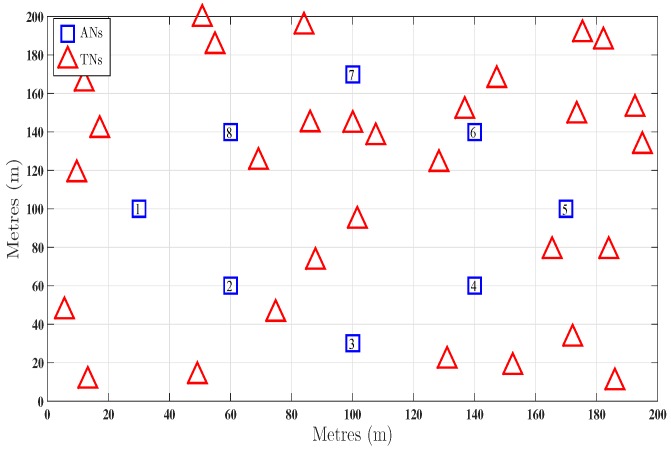
Network deployment with 30 target nodes (TNs) positioned at random unknown locations and 8 anchor nodes (ANs) at fixed known locations.

In this section, we report simulation results which evaluate the performance of the proposed techniques. A 200 m × 200 m network is considered with subsets of 8 ANs at fixed and known positions. To represent a realistic scenario the target nodes are taken at random locations. The parameters considered are given in Table 1 and the network deployment is shown in Figure 1.

**Figure 2:**
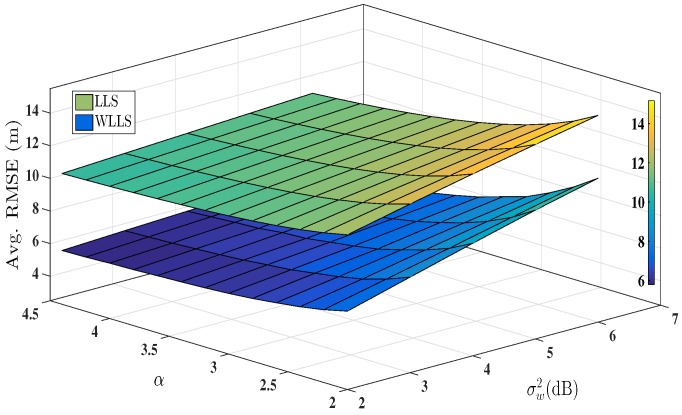
Performance comparison between linear least squares (LLS) and weighted linear least squares (WLLS) for hybrid angle of arrival (AoA)-received signal strength (RSS) measurement. $\sigma_{m}^{2}=4^{0}$, $\mathrm{ANs}=\left[1-8\right]$, $\alpha_{i}=2.5\;\forall \;i,$ ℓ=2500.

In Figure 2, using Monte Carlo simulation, the Avg. RMSE is plotted obtained with LLS approach and WLLS approach for localisation using hybrid AoA-RSS measurements. The noise in angle estimates is kept fixed at $\sigma_{m}^{2}=4$ while the shadowing noise is incremented gradually. Also, the PLE values are incremented from 2 to 4.5. The average RMSE is plotted across different values of shadowing noise variance and PLEs. It is evident from the Figure 2 that WLLS approach (based on the noise covariance matrix) has a better accuracy than LLS approach and showing around 25%–30% better results in terms of Avg. RMSE.

**Figure 3:**
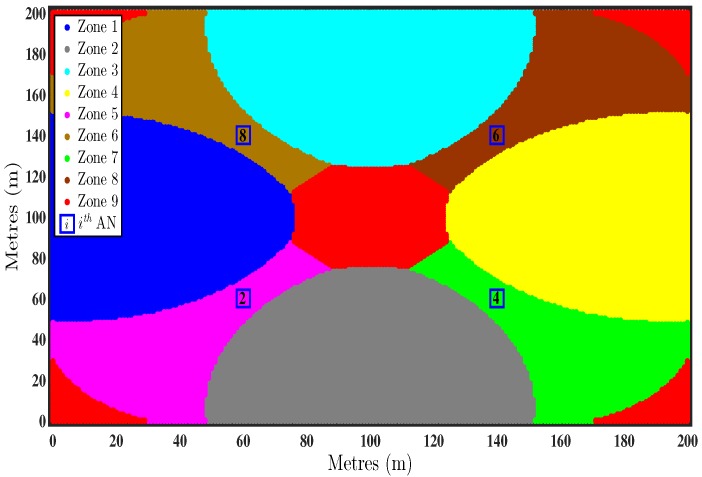
Division of network into different zone based on the theoretical mean square error (MSE). $\mathrm{ANs}=\left[2,4,6,8\right]$, $\alpha_{i}=2.5\;\forall \;i.$

In Figure 3, the network is divided into different zone. Each zone have its own optimal subset of ANs. Each color represents a different combination of ANs. It is evident from Figure 3 that using all ANs for localisation does not show minimum error for all TNs. The optimal combinations of ANs for the zones shown in Figure 3 are given in Table 2.

**Figure 4:**
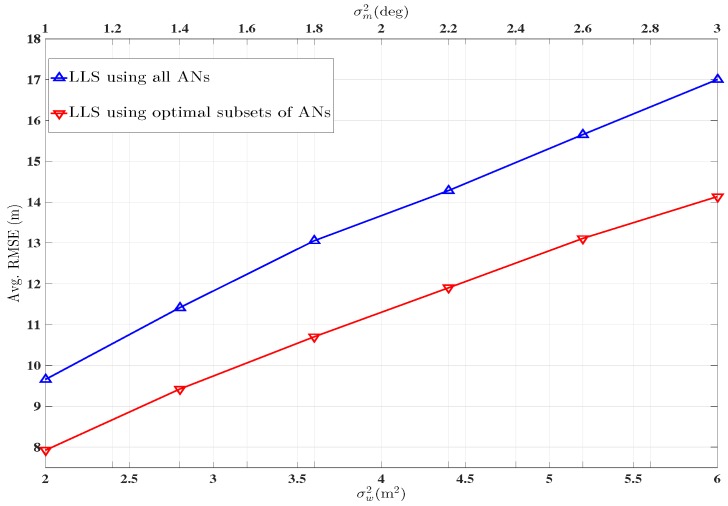
Performance comparison in terms of Avg. RMSE, using optimal subsets of ANs and using all ANs simultaneously. $\mathrm{ANs}=\left[2,4,6,8\right],$ ℓ=1000, $\alpha_{i}=2.5\;\forall \;i.$

For the configuration given in Figure 1, the network is divided into different zones, shown in Figure 3, using the theoretical MSE given by Equation (27). The blue and the red curves in Figure 4 represent the performance of the unbiased LLS algorithm using all available ANs and the subsets of ANs determined by the first step of the two step optimal AN selection scheme, respectively. In Figure 4, the Avg. RMSE is for all 30 TNs is plotted against shadowing noise variance on lower and angle noise variance on upper *x*-axis.

**Figure 5:**
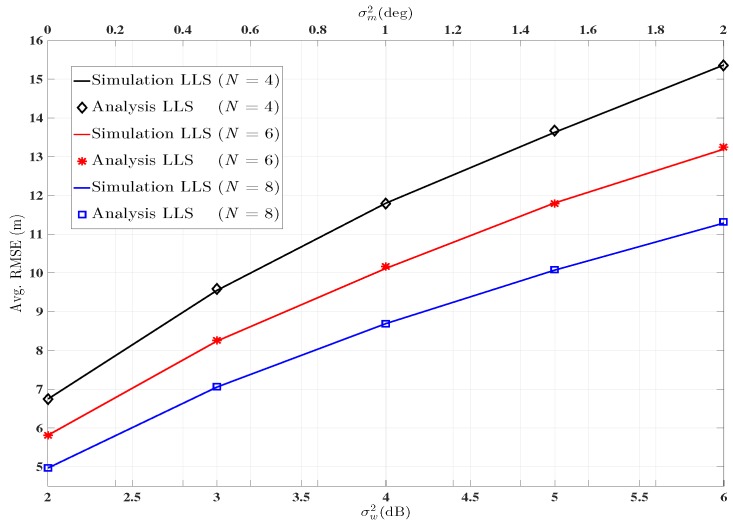
Performance evaluation via theoretical MSE expression and simulation for LLS. ANs= $\left[\left(2,4,6,8\right)\right.$, $\left(1,2,3,5,6,7\right),$ $\left.\left(1-8\right)\right]$, ℓ=1500, $\alpha_{i}=2.5\;\forall \;i$.

The theoretical MSE is used to demonstrate the system’s performance. In Figure 5, the curves represent the Avg. RMSE obtained by Monte Carlo simulation using different number of ANs. The markers represent the Avg. RMSE using the theoretical MSE expression derived in Section 4. Both performances are plotted across shadowing and angle noise variance which are taken across lower and upper *x*-axis. Since, the markers coincide with the curves, which shows the accurate prediction of the systems performance and which also proves that the mathematical derivation of the theoretical MSE expression in Section 4 is correct.

**Figure 6:**
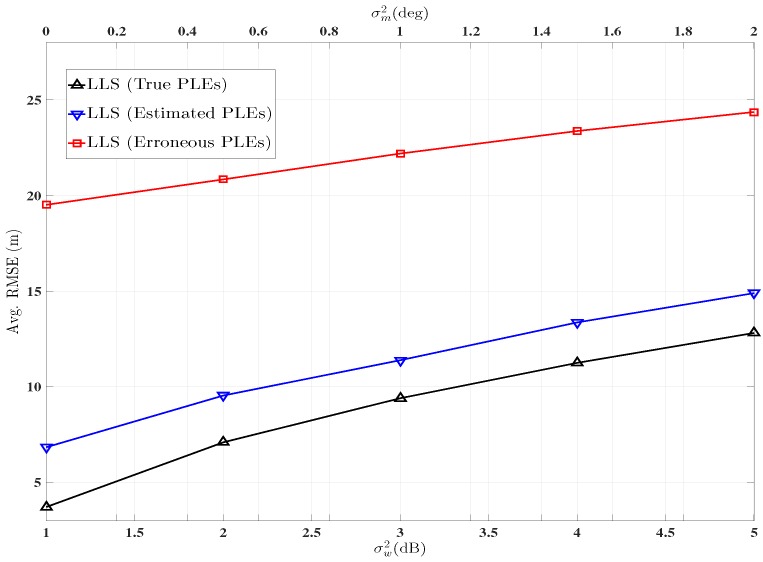
Avg. RMSE comparison using estimated PLEs and true PLE’s. $\mathrm{ANs}=\left[1-8\right]$, ℓ=2000, *τ* = 1, ξ=2, $\Delta_{0}=0.5,$ v=10, $\alpha_{i}\in U\left[2,\;5\right],$ $\alpha_{0}\in U\left[2,\;5\right],$ $\sigma_{p}=0.2.$

Figure 6 demonstrates the performance of the hybrid AoA-RSS signal model based on LLS approach when the PLE vector is estimated via the generalised pattern search. Each AN-TN link is associated with a different PLE, which is taken at random between 2–5. LLS using erroneous PLEs is also plotted for comparison, i.e., ${\bar{\alpha }}_{i}=\alpha_{i}+p_{i}$ where ${\bar{\alpha }}_{i}$ is the erroneous PLE and $p_{i}$ is the error associated with true PLE $\alpha_{i}$. $p_{i}$ is considered to be zero mean Gaussianly distributed random variable with the standard deviation $\sigma_{p_{i}}$ i.e., $p_{i}∼N\left(0,\sigma_{p_{i}}^{2}\right)$. For this simulation $\sigma_{p_{i}}=0.2$ is considered. It is observed that even such a small error in the PLE vector produces considerable error in the final estimate of the location of TN, while localisation using estimated PLEs produce considerably better estimates.

**Figure 7:**
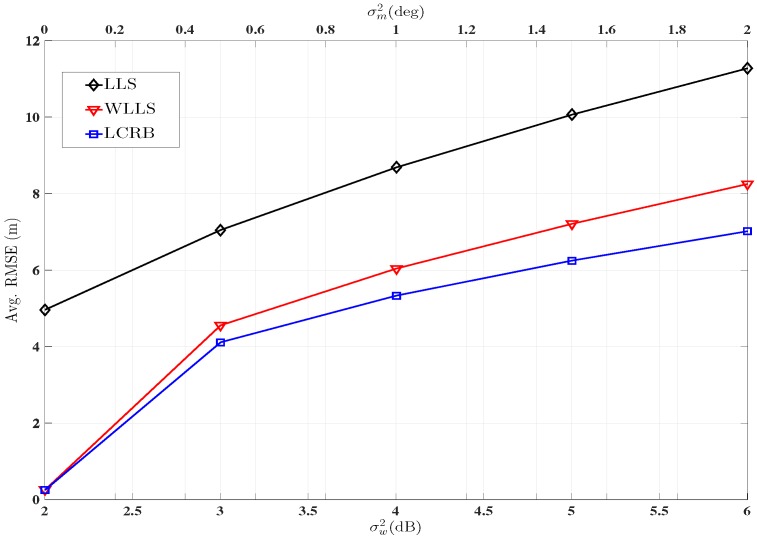
Performance comparison between LLS, WLLS and LCRB using hybrid AoA-RSS measurements. $\mathrm{ANs}=\left[1-8\right]$ $\alpha_{i}=2.5\;\forall \;i,$ ℓ=2000.

Comparison of the LLS, WLLS with its corresponding LCRB for the AoA-RSS measurement model is given in Figure 7. In this case, the PLE is kept fixed at 2.5. The Avg. RMSE of all TNs is plotted across both noise variance. Again it can be seen from Figure 7 that the WLLS outperforms the LLS model and that LCRB tightly bounds WLLS.

## 8. Conclusions

This paper focuses on hybrid localisation using AoA-RSS measurements. An unbiased LLS estimator is proposed and improvements to the LLS model are achieved by proposing a WLLS algorithm and by developing a methodology for optimal AN selection. A PLE estimation technique using the generalised pattern search is also proposed and a closed form MSE expressions for LLS is derived. Further analysis is done and LCRB is derived that tightly bounds WLLS solution.

It is demonstrated via simulation that the WLLS algorithm performs with 25%–30% better accuracy than the LLS algorithm while the performance is further enhanced with the optimal AN selection. It is also observed that the MSE expression accurately predicts the performance of the LLS technique. Furthermore, the generalised pattern search algorithm considerably improves the performance by accurately estimating the PLEs. Finally, it is shown that the LCRB derived, tightly bound the performance of WLLS estimator. The proposed algorithms are suitable for a number of applications including first aid responders, logistics and fault and fire detection.

## Figures and Tables

**Table 1:** Parameters description.

S.No	Symbol	Description
1	$\sigma_{m}^{2}$	Angle noise variance
2	$\sigma_{w}^{2}$	Shadowing noise variance
3	$\alpha_{i}$	PLE associated with *i*th link
4	$\alpha_{0}$	Initial PLE assumption (for initialising GenPS)
5	$\sigma_{p}$	Standard deviation of erroneous PLE
6	${▵}_{0}$	Initial step size in GenPS
7	${▵}_{k}$	Step size at *k*th iteration
8	*ξ*	Step size indicator in GenPS
9	*τ*	Stopping criteria for GenPS
10	*ℓ*	Number of iterations

**Table 2:** Optimal combinations of ANs for zones shown in Figure 3.

Zones	Optimal AN Combination
Zone 1	i=2,8
Zone 2	i=2,4
Zone 3	i=6,8
Zone 4	i=4,6
Zone 5	i=2,4,8
Zone 6	i=2,6,8
Zone 7	i=2,4,6
Zone 8	i=4,6,8
Zone 9	i=2,4,6,8

## Abbreviations

The following abbreviations are used in this manuscript:

AN	Anchor node
TN	Target Node
AoA	Angle of Arrival
RSS	Received Signal Strength
ToA	Time of Arrival
MSE	Mean Squares Error
Avg. RMSE	Average Root Mean Squares Error
LLS	Linear Least Squares
WLLS	Weighted Linear Least Squares
PLE	Path-loss Exponent
LCRB	Linear Cramer-Rao Bound
FIM	Fisher Information Matrix

## Appendix A. Derivation of Unbiasing Constant

The *i*th term of $\epsilon {\left(x\right)}_{i}=E_{w_{i},m_{i}}\left[\hat{b}{\left(x\right)}_{i}-b{\left(x\right)}_{i}\right],$ for $\hat{b}{\left(x\right)}_{i}$ in which $\delta_{i}$ is ignored, is given by,

$$\epsilon {\left(x\right)}_{i}=E_{w_{i},m_{i}}\left(x_{i}+d_{i}\exp\left(\frac{w_{i}}{\gamma \alpha_{i}}\right)\cos\left(\theta_{i}+m_{i}\right)-x_{i}-d_{i}\cos\theta_{i}\right)\;\mathrm{for}\;i=1,\ldots ,N$$

Using sum difference formula $\cos\left(\theta_{i}+m_{i}\right)=\cos\left(\theta_{i}\right)\cos\left(m_{i}\right)-\sin\left(\theta_{i}\right)\sin\left(m_{i}\right)$.

$$\begin{array}{cc} \epsilon {\left(x\right)}_{i}= & \left(d_{i}E_{w_{i}}\left[\exp\left(\frac{w_{i}}{\gamma \alpha_{i}}\right)\right]\right)\left(\cos\theta_{i}E_{m_{i}}\left[\cos m_{i}\right]-\sin\theta_{i}E_{m_{i}}\left[\sin m_{i}\right]\right)-d_{i}\cos\theta_{i} \end{array}$$

Taking the expectations $E_{w_{i}}\left[\exp\left(\frac{w_{i}}{\gamma \alpha_{i}}\right)\right]=\exp\left(\frac{\sigma_{w_{i}}^{2}}{\gamma \alpha_{i}}\right),$ $E_{m_{i}}\left[\cos m_{i}\right]=\exp\left(-\frac{\sigma_{m_{i}}^{2}}{2}\right)$ and $E_{m_{i}}\left[\sin m_{i}\right]=0,$ we obtain

$$\begin{array}{cc} \epsilon {\left(x\right)}_{i} & =d_{i}\cos\theta_{i}\left(\exp\left(-0.5\sigma_{m_{i}}^{2}+\frac{\sigma_{w_{i}}^{2}}{2{\left(\gamma \alpha_{i}\right)}^{2}}\right)-1\right) \end{array}$$

similarly the *i*th term of $\epsilon {\left(y\right)}_{i}=E_{w_{i},m_{i}}\left[\hat{b}{\left(y\right)}_{i}-b{\left(y\right)}_{i}\right]$ can be reduced to

$$\epsilon {\left(y\right)}_{i}=d_{i}\sin\theta_{i}\left(\exp\left(-0.5\sigma_{m_{i}}^{2}+\frac{\sigma_{w_{i}}^{2}}{2{\left(\gamma \alpha_{i}\right)}^{2}}\right)-1\right)$$

## Appendix B. Derivation of FIM

Derivation of Equation (38): Taking derivative of Equation (22) with respect to *x*

(B1) $$\frac{\partial C{\left(x\right)}_{{}_{ii}}}{\partial x}=\frac{\partial }{\partial x}\left(\frac{d_{i}^{2}}{2}\right)\kappa_{i}+\frac{\partial }{\partial x}\left(\frac{d_{i}^{2}}{2}\cos2\theta_{i}\right){\bar{\kappa }}_{i}-\frac{\partial }{\partial x}{\left(d_{i}\cos\theta_{i}\right)}^{2}$$

Utilising product rule

$$\begin{array}{cc} \frac{\partial C{\left(x\right)}_{{}_{ii}}}{\partial x}= & \left(x-x_{i}\right)\kappa_{i}+\left(\frac{d_{i}^{2}}{2}\frac{\partial }{\partial x}\cos2\theta_{i}+\cos2\theta_{i}\frac{\partial }{\partial x}\frac{d_{i}^{2}}{2}\right){\bar{\kappa }}_{i}-\frac{\partial }{\partial x}{\left(d_{i}\cos\theta_{i}\right)}^{2} \end{array}$$

(B2) $$\begin{array}{cc} = & \left(x-x_{i}\right)\kappa_{i}+\left(d_{i}^{2}\sin2\theta_{i}\frac{\partial }{\partial x}\theta_{i}+\cos2\theta_{i}\left(x-x_{i}\right)\right){\bar{\kappa }}_{i}-\frac{\partial }{\partial x}{\left(d_{i}\cos\theta_{i}\right)}^{2} \end{array}$$

(B3) $$\begin{array}{cc} = & \left(x-x_{i}\right)\kappa_{i}+\left(\left(y-y_{i}\right)\sin2\theta_{i}+\cos2\theta_{i}\left(x-x_{i}\right)\right){\bar{\kappa }}_{i}-2\left(x-x_{i}\right) \end{array}$$

This is the required solution and it is obtained from Equation (B2) after taking the following derivatives $\frac{\partial }{\partial x}\theta_{i}=\frac{\left(y-y_{i}\right)}{d_{i}^{2}},$ $\frac{\partial }{\partial x}\left(\frac{d_{i}^{2}}{2}\right)=\left(x-x_{i}\right)$ and $\frac{\partial }{\partial x}{\left(d_{i}\cos\theta_{i}\right)}^{2}=2\left(x-x_{i}\right).$

Derivation of Equation (39): Taking derivative of Equation (22) with respect to *y*.

(B4) $$\frac{\partial C{\left(x\right)}_{{}_{ii}}}{\partial y}=\frac{\partial }{\partial y}\left(\;\frac{d_{i}^{2}}{2}\right)\;\kappa_{i}+\frac{\partial }{\partial y}\left(\frac{d_{i}^{2}}{2}\cos\;2\theta_{i}\right){\bar{\kappa }}_{i}-\frac{\partial }{\partial y}{\left(d_{i}\cos\theta_{i}\right)}^{2}$$

$$\begin{array}{cc} \frac{\partial C{\left(x\right)}_{{}_{ii}}}{\partial y}= & \left(y-y_{i}\right)\kappa_{i}+\left(\frac{d_{i}^{2}}{2}\frac{\partial }{\partial y}\cos2\theta_{i}+\cos2\theta_{i}\frac{\partial }{\partial y}\frac{d_{i}^{2}}{2}\right){\bar{\kappa }}_{i}-\frac{\partial }{\partial y}{\left(d_{i}\cos\theta_{i}\right)}^{2} \end{array}$$

(B5) $$\begin{array}{cc} = & \left(y-y_{i}\right)\kappa_{i}+\left(-d_{i}^{2}\sin2\theta_{i}\frac{\partial }{\partial y}\theta_{i}+\cos2\theta_{i}\left(y-y_{i}\right)\right){\bar{\kappa }}_{i}-\frac{\partial }{\partial y}{\left(d_{i}\cos\theta_{i}\right)}^{2} \end{array}$$

(B6) $$\begin{array}{cc} = & \left(y-y_{i}\right)\kappa_{i}+\left(\cos2\theta_{i}\left(y-y_{i}\right)-\sin2\theta_{i}\left(x-x_{i}\right)\right){\bar{\kappa }}_{i} \end{array}$$

Equation (B6) is obtained from Equation (B5) by using the following derivatives $\frac{\partial }{\partial y}\theta_{i}=\frac{\left(x-x_{i}\right)}{d_{i}^{2}}$ and $\frac{\partial }{\partial y}{\left(d_{i}\cos\theta_{i}\right)}^{2}=0.$

Derivation of Equations (40) and (41) is similar, other than the fact that *x* variable is replaced by *y*.

Derivation of Equation (42): Taking derivative of Equation (24) with respect to *x*.

(B7) $$\frac{\partial C{\left(xy\right)}_{{}_{ii}}}{\partial x}\;=\;\frac{\partial }{\partial x}\left(d_{i}^{2}\cos\theta_{i}\sin\theta_{i}\right)\left[{\bar{\kappa }}_{i}-1\right]$$

which can written after using product rule as

(B8) $$\begin{array}{ll} \frac{\partial }{\partial x}\left(d_{i}^{2}\cos\theta_{i}\sin\theta_{i}\right) & =d_{i}^{2}\cos\theta_{i}\frac{\partial }{\partial x}\sin\theta_{i}\;+\;\sin\theta_{i}\frac{\partial }{\partial x}d_{i}^{2}\cos\theta_{i}\\ & =d_{i}^{2}\cos\theta_{i}\frac{\partial }{\partial x}\sin\theta_{i}\;+\;d_{i}^{2}\sin\theta_{i}\frac{\partial }{\partial x}\cos\theta_{i}\;+\;\sin\theta_{i}\cos\theta_{i}\frac{\partial }{\partial x}\left(d_{i}^{2}\right)\\ & =-d_{i}^{2}\cos\theta_{i}\left(\cos\theta_{i}\frac{\left(y-y_{i}\right)}{d_{i}^{2}}\right)+d_{i}^{2}\sin\theta_{i}\left(\sin\theta_{i}\frac{\left(y-y_{i}\right)}{d_{i}^{2}}\right)\\ & \;\;\;+\sin\theta_{i}\cos\theta_{i}\left(2\left(x-x_{i}\right)\right) \end{array}$$

Replacing Equation (B8) in Equation (B7) we get Equation (42).

Derivation of Equation (43): Taking the derivative of Equation (24) with respect to *y*.

(B9) $$\frac{\partial C{\left(xy\right)}_{{}_{ii}}}{\partial y}\;=\;\frac{\partial }{\partial y}\left(d_{i}^{2}\cos\theta_{i}\sin\theta_{i}\right)\left[{\bar{\kappa }}_{i}-1\right]$$

The derivatives in Equation (B9) is given as

(B10) $$\begin{array}{ll} \frac{\partial }{\partial y}\left(d_{i}^{2}\cos\theta_{i}\sin\theta_{i}\right) & =d_{i}^{2}\cos\theta_{i}\frac{\partial }{\partial y}\sin\theta_{i}+\sin\theta_{i}\frac{\partial }{\partial y}d_{i}^{2}\cos\theta_{i}\\ & =\cos^{2}\theta_{i}\left(x-x_{i}\right)-\sin^{2}\theta_{i}\left(x-x_{i}\right)+2\sin\theta_{i}\cos\theta_{i}\left(y-y_{i}\right) \end{array}$$

Replacing Equation (B10) in Equation (B9) we get Equation (43).
